# The clinical results and function of the intersegmental disc after posterior-only partial corpectomy for lumbar burst fractures

**DOI:** 10.1097/MD.0000000000040610

**Published:** 2024-11-29

**Authors:** Pengzhan Liang, Xiaodong Chen, Gao Shu, Haibo Zhao, Jinquan Lai, Linbo Jiang, Xuejun Yang

**Affiliations:** a Shenzhen Luohu District TCM Hospital, Shen Zhen, China.

**Keywords:** disc degeneration, lumbar fracture, posterior approach, subtotal corpectomy, vertebral height

## Abstract

We treated the burst spinal fracture with posterior subtotal corpectomy and reconstruction. In some cases, the endplate and the adjacent disc can be preserved during the operation. The adjacent disc retained its mobility after the removal of the posterior pedicle screws. This study evaluated the clinical results and function of the intersegmental caudal disc after the removal of posterior pedicle screws for lumbar burst fractures. The study analyzed retrospectively 36 patients with acute burst traumatic lumbar fractures who underwent posterior partial subtotal corpectomy and reconstruction with preservation of the inferior endplate of the fractured vertebral body and the adjacent caudal disc, and sequential removal of the posterior pedicle screw 1 year after the second surgery from March 2015 to December 2021. All patients were followed for approximately 1 year after pedicle screw removal. Demographic data, anterior vertebral body height, local kyphosis, motion, caudal disc degeneration, and clinical outcomes were evaluated. After removal of the posterior pedicle screws, the intersegmental disc retained a range of motion of 10.55 ± 5.58°, and the disc degeneration was graded by Pfirrmann criteria from 2.21 ± 1.15 before first surgery to 3.18 ± 1.46 at last follow-up after second surgery. There were 2 cases of superficial wound infection, and 5 cases of postoperative neuralgia recovering after 3 months. Anterior fusion was achieved, although postoperative subsidence of the mesh was observed in 6 cases and screw loosening in 9 cases. Posterior-only partial subtotal corpectomy with preservation of the adjacent caudal disc not only achieved a good clinical effect, but also preserved intersegmental caudal disc function after removal of posterior pedicle screws approximately 1 year later. This technique is a promising alternative for cases in which the endplate obviates injury.

## 1. Introduction

Surgery is generally recommended for patients with spinal fractures, neurological deficits, or severe instability.^[1–8]^ It is important to adopt short-segment pedicle screw fixation when managing unstable lumbar fractures to avoid sacrificing mobile segments and adjacent discs. Posterior internal fixation failures and post-traumatic kyphosis due to a lack of anterior column support regarding long or short fixation have always been reported when the anterior spinal columns cause deficient support.^[9,10]^ Anterior reconstruction plays an important role in burst unstable fractures by the anterior approach,^[2]^ posterior approach^[3–5]^ or associated approach^[6–8]^ to achieve ultimate spinal stabilization by successful bone healing.

Posterior short internal fixation with anterior column reconstruction has become the mainstream treatment for burst spinal fractures. Meanwhile, short internal fixation obviates damage to adjacent mobile segments. In 2006, Hunt et al^[3]^ described a technique for inserting an expandable cage via a posterior extra-cavitary approach while protecting the segmental nerves. Currently, this technique has evolved and is widely used for spinal fractures^[4,5]^, Kummell’s disease^[11]^, neoplasm, and osteomyelitis.^[12]^

Clinically, in some cases, one vertebral endplate always escapes injury and remains intact in the burst fractured vertebral body; therefore, it is desirable to preserve the adjacent disc in fixed segments. Intersegmental spinal mobility can be retained after the removal of the posterior pedicle screws. For example, subtype A3 injury based on the AO classification system^[13]^ always shows a single endplate fracture with any involvement of the posterior vertebral wall and spinal canal; therefore, another intact endplate can act as a footstone for cage reconstruction. We retrieved intersegmental spinal mobility in the fixed segments after removal of the posterior pedicle fixation if we preserved the intact vertebral endplate and the adjacent disc. It sounds similar to the subtotal corpectomy technique^[3–5]^ which excises the cephalic and caudal discs and endplates of the fractured vertebral body. We found that a technique that preserves the caudal disc in spinal fractures has been used in some reports.^[7,8]^ Both studies reported a case that underwent posterior stabilization and mono-segmental anterior reconstruction with preservation of the intact inferior endplate and the adjacent caudal disc. As we know, it’s first time to pay attention to report the technique to perverse the adjacent discs.

## 2. Methods

### 2.1. Patient populations

The study participants were 36 patients (22 males, 14 females; mean age 38.4 [range, 21–55] years) with acute burst traumatic lumbar fractures who underwent pedicle screw removal after successful posterior-only corpectomy and reconstruction between March 2015 and December 2021 at our hospital. The study inclusion criteria were as follows: patients with a single burst lumbar fracture; patients who underwent one-stage posterior subtotal corpectomy and mesh reconstruction with preservation of the caudal vertebral endplates and adjacent discs; short segmental fixation at one level above and below the affected level; 1 year later, the patients underwent a second surgery to remove the posterior fixations; and patients were followed up both clinically and radiologically for more than 1 year after implant removal surgery. The exclusion criteria were as follows: pathologic lesions and intraoperative excision of caudal vertebral endplates and adjacent discs; Long segmental fixation of more than one level above and below the affected level; no removal of the pedicle screws or beyond the terminal time (15 months); and loss of follow-up. According to the AO (Association for the Study of Internal Fixation) system,^[13]^ 19 fractures were classified as type A3, 12 as B2, and 5 as C. The mean interval from first fracture surgery to second implant removal and second implant removal to final follow-up were 12.7 (range, 10–15) months and 19.8 (range, 13–32) months respectively. Nine fractures were located at L1, 12 at L2, 7 at L3, and 4 at L4. (Table 1).

**Table 1:** Summarized data of patients

Mean age in years (range)	38.4 (21–55)
Male sex, n (%)	22 (72)
mean follow-up in month (range)	
Between fracture surgery and implant removal	12.7 (10–15)
Between implant removal and final follow-up	19.8 (13–32)
The fracture type, n (%)	
A3	19 (53)
B2	12 (33)
C	5 (14)
Neurological injuries by Frankle, n (%)	
Grade A	3 (8)
Grade B	6 (17)
Grade C	5 (14)
Grade D	6 (17)
Grade E	14 (39)
Cauda equina	2 (6)
Fracture level, n (%)	
L1	9 (25)
L2	12 (33)
L3	7 (19)
L4	4 (11)
Mean blood loss (mL, range)	750 (400–1500)
Mean operation time (min, range)	215 (160–350)
Op complications, n (%)	9 (25)
Cerebrospinal fluid leakage	2 (6)
Nerve root injury	5 (14)
Superficial infection	2 (6)
IF complication at L-FU, n (%)	15 (42)
Subsidence	6 (17)
Screws loosen	9 (25)

IF = internal fixation, L = lumbar, L-FU = last follow-up, OP = operation.

### 2.2. Management

The patients were placed in the prone position on a radiolucent spine table after general anesthesia. A posterior straight midline incision centered on the affected level was created to expose the laminae one level above and below the affected level. The pedicle screws were then introduced. Facetectomy and laminectomy were then performed on the side of neurological injury or severe comminuted vertebral body fracture for decompression and stabilization. The contralateral laminae and facet joints were retained as bone graft beds for the posterior fusion. The dural sac was stitched if lacerated. Partial superior corpectomy and cranial discectomy were performed via a posterior approach, and the intact inferior endplate of the fractured vertebral body on which the mesh could be located was preserved. The screws on the contralateral side were distracted axially with a contoured rod to restore segmental height and realign the spinal columns. The height between the inferior endplates of the adjacent cephalad vertebrae and the preserved fractured vertebrae was measured to determine the correct length of the titanium mesh.

The thecal sac and exiting and traversing nerve roots were gently retracted and protected using a retractor. Titanium meshes packed with autologous bone harvested from the resected bony structures were inserted between the neural elements in a lengthwise fashion using a screwdriver to control torsion and then the cage was flipped into the proper position. Posterior fusion was performed between the contralateral vertebral plates of the fractured and adjacent cephalad vertebrae using allografts and autografts. The incision was closed after the drain placement. Ambulation was started after 3 days with a Thoraco-Lumbo-Sacral Orthosis for 8 to 12 weeks. The posterior screws were removed approximately 1 year after the primary surgery.

### 2.3. Radiographic and functional outcome evaluation

**Figure 1.**
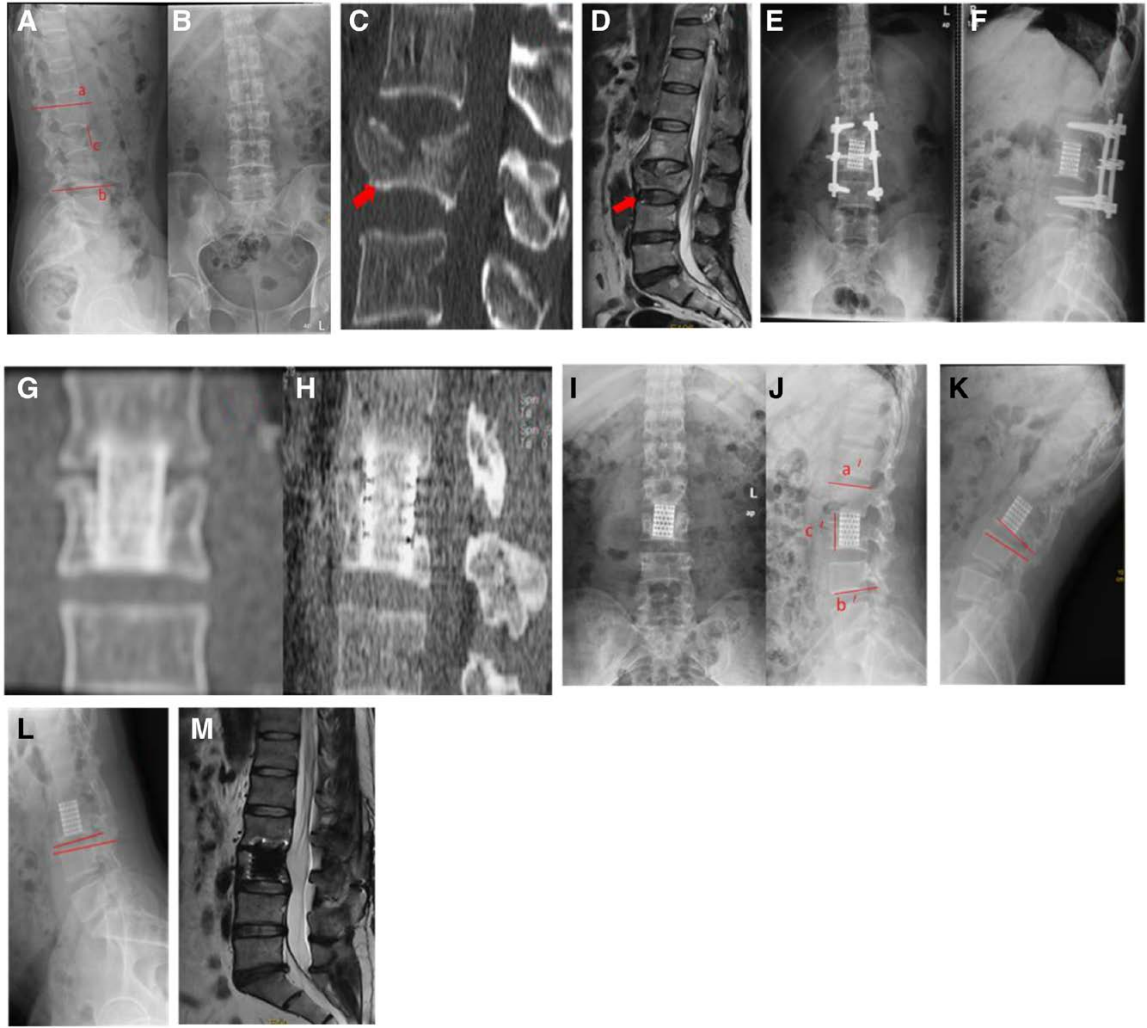
Imaging findings in a 32-year-old man who had a burst fracture at the third lumbar vertebra with a neurological injury graded D by Frankel grade on admission. (A, B) Preoperative anteroposterior and lateral films. local lordosis (L2–L4, red line a and b) was 0º. The vertebral height (red line c) was 25.7 mm. (C) A preoperative computed tomography scan. The inferior endplate at L3 (red bold arrow) was intact. (D) A preoperative magnetic resonance image. The caudal disc (red bold arrow) was assessed as grade 2 by Pfirrmann. (E, F) Postoperative anteroposterior and lateral films. (G, H) Computed tomography scans obtained at the last follow-up showed successful bone fusion after removal of internal fixation. (I, J) Radiographs obtained at the last follow-up showed local lordosis (L2–L4, red line a′ and b′) was −10º. The vertebral height (red line c′) was 38.4 mm. (K, L) Flexion-extension radiographs obtained at the last follow-up showed the motion of disc was 10º. (M) Magnetic resonance imaging performed at the last follow-up showed that the disc degeneration was same as the preoperative grade.

Radiological assessments were performed using radiographs and magnetic resonance (MR) images of the injured areas. Local kyphosis (LK) was measured from the superior endplate of the vertebra cephalad to the fractured vertebra to the inferior endplate of the vertebra caudal to the fractured vertebra. The extent of intersegmental disc degeneration was assessed on midsagittal T2-weighted MR images according to Pfirrmann criteria^[14]^ as grade I (score 1), grade II (score 2), grade III (score 3), grade IV (score 4), or grade V (score 5). Vertebral height (VH) was measured between the anterior edges of the inferior endplate of the fractured vertebra and the intact cephalic vertebra. The ROM of disc was achieved from dynamic X-rays (Fig. 1). Radiological measurements were obtained and recorded in triplicate on a PACS workstation by 3 experienced attendants. The investigators calculated the mean values for each parameter. Clinical results were assessed using the mean visual analog scale (VAS) score. Patients were asked to rate their current level of back pain on a scale of 0 to10 (0, no pain; 10, worst pain unimaginable).

### 2.4. Statistical analysis

The data for continuous variables are summarized as means and standard deviations. A paired *t* test was used to examine differences between the 2 groups. All statistical analyses were performed using SPSS version 19.0 software (IBM Corp., Armonk). Statistical significance was set *P* value < .05.

## 3. Results

### 3.1. Vertebral height and local kyphosis

The average VH increased from 21.58 ± 5.13 mm before surgery (BS) to 40.59 ± 7.87 mm after surgery (AS), with a mean restoration of 19.01 mm. The mean VH was 38.27 ± 6.27 mm at pre-implant removal (Pre-IR) and 37.17 ± 6.55 mm at post-implant removal (post-IR). At the final follow-up (F-FU) visit, the average VH was 35.85 ± 6.43 mm. No significant differences were observed in the demographic data between these groups (AS vs pre-IR, pre-IR vs post-IR, and post-IR vs F-FU), except for the other 2 groups (BS vs AS) (Table 2).

**Table 2:** Radiographic measurement, disc degeneration and visual analog scale in course of follow-up

Parameter	BS	AS	Pre-IR	Post-IR	F-FU
VH (mm)	21.58 ± 5.13	40.59 ± 7.87	38.27 ± 6.27	37.17 ± 6.55	35.85 ± 6.43
*P* value		<.05	>.05	>.05	>.05
LK (°)	−25.33 ± 7.93	15.27 ± 5.63	11.78 ± 4.46	9.38 ± 4.58	8.87 ± 3.86
*P* value		<.05	>.05	>.05	>.05
ROM (°)					10.55 ± 5.58
Pfirrmann	2.21 ± 1.15				3.18 ± 1.46
*P* value					>.05
VAS	6.57 ± 1.75	2.13 ± 1.52	1.83 ± 1.66		1.47 ± 1.23
*P* value		<.05	>.05		>.05

Statistical comparison between BS and AS, AS and Pre-IR, Pre-IR and Post-IR, Post-IR and F-FU at VH and LK, between BS and AS, AS and Pre-IR, Pre-IR and F-FU at VAS.

° = degree, AS = after surgery, BS = before surgery, F-FU = final follow-up, LK = local kyphosis, mm = millimeter, post-IR = post-implant removal, Pre-IR = pre-implant removal, ROM = range of motion, VAS = visual analog scale, VH = vertebral height.

The average LK increased from −25.33 ± 7.93° BS to 15.27 ± 5.63° AS, with a mean restoration of 40.6°. The average LK was 11.78 ± 4.46° at Pre-IR and 9.38 ± 4.58° at Post-IR; therefore, the loss of reduction was 2.4°. The average LK was 8.87 ± 3.86° at the F-FU, indicating a loss of reduction of 0.51° in comparison with post-IR. No significant differences were observed in the demographic data between these groups (AS vs pre-IR, pre-IR vs post-IR, and post-IR vs F-FU), except for the other 2 groups (BS vs AS) (Table 2).

### 3.2. Disc motion and degeneration

Range of motion (ROM) of the inter-segmental disc was 10.55 ± 5.58° at the last follow-up on flexion-extension radiography after removal of the posterior fixation. The degeneration graded by Pfirrmann criteria of inter-segmental disc was 2.21 ± 1.15 BS, which deteriorated to 3.18 ± 1.46 at F-FU (Table 2).

### 3.3. Clinical outcomes and complications

The mean VAS score for back pain improved from 6.57 ± 1.75 BS to 2.13 ± 1.52 AS. The VAS scores at pre-IR and F-FU were 1.83 ± 1.66 and 1.47 ± 1.23 respectively. No significant differences were observed in the demographic data between these groups (AS vs pre-IR, and pre-IR vs F-FU), except for the other 2 groups (BS vs AS) (Table 2).

Postoperative subsidence of the prosthetic cage at the adjacent endplates was observed in 6 cases and screw loosening in 9 cases before implant removal, although fusion was accomplished before implant removal. There were 2 superficial wound infection, 2 cerebrospinal fluid leakage, 5 postoperative neuralgia but recovering after 3 months. 22 patients were identified to have a partial neurological deficit according to the Frankel grade.^[15]^ Postoperatively, 2 patients improved from grade B to grade D, 1 from B to C, 3 from B to E, 4 from C to E, 1 from C to D, and 6 from D to E. Two patients with cauda equina suffered irreversible damage. The remaining 14 patients remained neurologically intact during the follow-up. The mean blood loss and operation time were 750 mL and 215 seconds, respectively (Table 1).

## 4. Discussion

The goal for the treatment of lumbar burst fractures is to restore vertebral height, alignment, and lordosis, decompress the spinal canal, and restore spine stability. The algorithm for lumbar fractures should be developed according to the fracture type, stability, and neurologic status.^[1,13]^ A burst unstable fracture causes a large bony defect within the fractured vertebra, and is accompanied by interspinous ligament injury and local kyphosis in most cases. A large anterior defect contributes to high levels of stress on the posterior construction. Without anterior support, posterior instrumentation is at risk of failure.^[4–6]^ Anterior implantation and reconstruction can decrease the load on the posterior fixation system, avoiding hardware loosening, maintain the interbody height, and preserve regional lumbar alignment.

Therefore, it is important to select suitable cases for our technique of partial corpectomy. We strictly selected the right cases based on the AO spine thoracolumbar injury classification system. A3 injuries are vertebral fractures affecting a single endplate with any involvement of the posterior vertebral wall and spinal canal; therefore, another intact endplate, acting as a footstone, is a prerequisite for a subtotal corpectomy for cage reconstruction. Subtype B2 injuries demonstrate disruption of the posterior tension band with or without osseous involvement, which offers the possibility of an intact endplate that acts as a footstone to support cage reconstruction during surgery. Type C injuries are characterized by displacement beyond the physiological range of the cranial and caudal parts of the spinal column in any plane. If the injury involves only one disc and posterior tension band without osseous structures, or if posterior osseous involvement does not affect anterior support for cage reconstruction, it is suitable for inclusion in the study criteria. In our study, we included subtypes A3, B2, and C with some prerequisites that an endplate is intact, the posterior bony structure maintains relative integrity for cage support, and the targeted disc that needs to be reserved does not visibly suffer from the injury.

There are some important details and differences between the techniques of modified partial corpectomy and subtotal corpectomy that have been reported. Based on our observations, the bony defect consistently occurs in the superior portion of the fractured vertebral body, so we just excise the defective superior partial vertebral body in this series distinguished with other corpectomy techniques that the most fractured body and cephalic and caudal disc were excised.^[4–6,16,17]^ We retained the inferior endplate of the fractured vertebrae and caudal disc. Meanwhile, posterior fusion with an allograft or autograft was only performed between the injured lamina and cephalic lamina and not at the caudal lamina. The subtotal corpectomy with cephalic and caudal discectomy requires excessive exposure of the exiting and traversing nerve roots to locate the cage; however, our technique did not require excessive exposure and stretching of the exiting and traversing nerve roots because we placed a shorter cage, which decreased nerve root injury. Five patients with postoperative neuralgia in our study recovered after 3 months.

Clinically, we did not persuade the patients to accept the removal of the internal fixation due to secondary trauma. If a patient wanted to remove the internal fixation, we informed them of the disadvantages of leaving the implants in situ, such as back pain, functional impairments, and implant-induced injury to the spine.^[18]^ Implant removal surgery has been widely used, especially among non-fusion patients in recent years, and the optimal time for removal of posterior internal fixation is approximately 1 year.^[18–21]^ We could not find these explanations; however, they are presented here. First, fusion was successfully accomplished about 1 year. Second, Patients who underwent implant removal within 12 months could obtain better clinical outcomes, especially in the ODI and EQ-5D, and patients who underwent implant removal within 12 months could also regain greater ROM of the fixed segments.^[22]^ Third, the ratio of spontaneous fusion of the articular process increases with time. According to the latest research by Ishihara M,^[23]^ the study investigated the process and morphology of thoracic bone fusion in patients who underwent percutaneous pedicle screws without bone grafting and found that the bone fusion rate in the thoracic spine was 54% after 3 postoperative years. We believe that a similar situation prevails in the lumbar region to decrease the disc mobility.

In our cases, there was a significant improvement in the anterior VH and LK after the surgery. Although VH and LK were slightly reduced after pre-implant removal, they did not affect clinical outcomes. Fortunately, the VH and LK were maintained until the last follow-up visit. The issues of VH and LK reductions are ubiquitous and have received considerable attention. Pellisé et al^[24]^ reported that one third of patients who underwent posterior 6-screw instrumented fixation had a 9° loss of correction in regional kyphosis. Prospective randomized studies by Chou et al^[21]^ and Božík et al^[25]^ identified a significant loss in mean postoperative LK. Leferink VJ^[26]^ suggested that the loss of correction in patients may be related to detachment of the soft tissues, decortication during preparation for arthrodesis, disruption of the supraspinous and interspinous ligaments, and shifting of the load of force back onto the anterior column. We believe that the intersegmental disc degeneration that degenerated from 2.21 ± 1.15 to 3.18 ± 1.4, grouped on Pfirrmann, and the subsidence of the mesh occurred in 6 cases, which were likely the causes of correction loss in our series.

Postoperatively, the neurological deficit of the 22 patients according to the Frankel grade obviously improved. We want to know which variable has an association with neurologic damage in thoracolumbar

burst fractures. Fracture level was an independent risk factor for neurological deficits. Yugue´ et al^[27]^ noted that the fracture level is a probable cause of this discrepancy because of variation in location and variable sensitivity to injury in different neurological structures. The canal encroachment ratio was most strongly associated with neurological deficits in traumatic mid and low lumbar fractures,^[27,28]^ so the patients with neurological deficits need direct decompression. A prospective and consecutive case series study^[29]^ shown New Injury Severity Score have a closer correlation with neurological deficit in thoracolumbar burst fractures. Early stabilization of the spine decreases the injury by improving respiratory function, shortening the duration of mechanical ventilation and intensive care unit stay and thus accelerating the recovery of neurological deficits.

The pathophysiology of segmental disc degeneration in spinal fractures remains controversial; however, there is a growing consensus that endplate fracture is an important factor responsible for the degeneration process. In 1998, Oner et al^[30]^ reported that the signal intensity of the discs was preserved, and most changes were related to morphological alterations in the disc space at a minimum of 18 months after injury. Choi et al^[31]^ found that endplate fractures accelerate the degeneration process. It has been verified by some researchers, both in humans^[32]^ and in animal models^[33]^ that endplate fracture can cause disc degenerative changes. Wang et al^[34]^ reported that disc degeneration occurred during the period of temporary fixation of the thoracolumbar spine for 9 to 22 months, and that the discs caudal to the fractured vertebra did not degenerate further on MRI. The adjacent disc was preserved based on an intact endplate, which again provides a theoretical foundation for our technique that preserves the caudal disc.

Although the caudal disc underwent some degree of degeneration, some activity was retained. On the above-mentioned efforts, we retrieved spinal intersegmental mobility about 11° after removal of internal fixation at final follow-up. Jeon et al^[18]^ reported that removal of pedicle screws in patients treated successfully for lumbar burst fractures contributed to the improvement in segmental motion angle observed at the 1-year and 2-year follow-up visits. Another study^[19]^ demonstrated that implant removal could restore mobility after posterior fracture fixation without the need for a bone graft in the thoracolumbar segment. It is an important protective factor for the adjacent disc biomechanically to retrieve one intersegmental lumbar mobility after removal of the posterior internal fixation. In a study of patients with burst fractures treated with pedicle screw fixation, Akbarnia et al^[35]^ found that 43 facet joint complexes that were not fused maintained physiological motion after instrumentation removal. Su et al^[36]^ observed an increase in spinal motion following removal of the pedicle screws after one-segment fusion. Similarly, a study by Toyone et al^[37]^ found an average range of motion of 12° in flexion/extension in the affected segments at 10-year follow-up in patients whose implants were removed within 1 year, followed by short-segment instrumentation without fusion surgery.

The clinical outcomes were good after hardware removal in our patients despite a slight loss of VH and LK. Jeon et al^[19]^ reported good mean VAS scores in the implant removal group at both 1-year and 2-year follow-up visits. Moreover, a meta-analysis^[38]^ showed no association between the degree of residual kyphosis and the severity of VAS pain scores or functional status. Good clinical outcomes have been associated with high fusion rates. We were concerned about the fusion rate because the caudally preserving disc allowed micromotion in the fusion contact surface under a bending load, which could obviate rigid fixation. This exactly follows the Biological Osteosynthesis (BO) principle that fractures do not require rigid fixation, but a low elastic modulus fixator. The caudally preserved disc allows the amount of micromotion to be flexible. In our study, the fusion rate was 100%, although fixation failure complications occurred in 15 cases, including cage subsidence in 6 cases and screw loosening in 9 cases, which were caused by the decreased rigidity of internal fixation resulting in excessive motion.

This study has some limitations that stem mainly from the lack of a control group for radiologic comparison of disc degeneration and range of motion and from the relatively small study group. MRI was not a routine examination, and ROM data were not obtained before surgery or during fixation. Furthermore, as in any retrospective study, there was uncertainty regarding the completeness and accuracy of the medical records and clinical information. Other socioeconomic and psychosocial factors that may have affected the clinical outcomes did not consider.

## 5. Conclusion

Posterior-only superior partial corpectomy with titanium mesh reconstruction and short-segment pedicle screw fixation is an important alternative to treat burst lumbar fractures with an intact inferior endplate, and retains the range of motion of adjacent caudal discs after removal of posterior pedicle screws 1 year later.

## Acknowledgments

We are very grateful to Shen Zhen Ping Le Orthopedics and Traumatology Hospital for their kind support with this study.

## Author contributions

**Data curation:** Gao Shu, Haibo Zhao, Jinquan Lai, Linbo Jiang.

**Funding acquisition:** Xuejun Yang.

**Project administration:** Pengzhan Liang.

**Resources:** Gao Shu.

**Software:** Xuejun Yang.

**Supervision:** Pengzhan Liang.

**Writing – original draft:** Pengzhan Liang.

**Writing – review & editing:** Xiaodong Chen.
